# Ophthalmic Surgery in Patients with Left Ventricular Assist Devices

**DOI:** 10.1155/2019/5097597

**Published:** 2019-03-20

**Authors:** George A. Dumas, Ayesha S. Bryant, Gwendolyn L. Boyd

**Affiliations:** University of Alabama at Birmingham, Department of Anesthesiology and Perioperative Medicine, Birmingham, Alabama, USA

## Abstract

The newer generation of left ventricular assist devices (LVADs) are commonly used as destination therapy; these devices have demonstrated improved outcomes and increased survival. Given the longer lifespan, it is not surprising that patients with LVADS are increasingly presenting with noncardiac, chronic diseases and interventions for their treatment. This includes ophthalmic procedures in patients with LVAD. There is a paucity of literature about the experiences and outcomes in this cohort of patients presenting for ophthalmologic surgery. Here we present a case series consisting of 7 patients with LVAD that underwent 10 ophthalmic surgeries. No adverse events including intraoperative hemodynamic instability or respiratory compromise occurred. All patients had an on-time discharge with no 30-day recidivism. Most patients underwent a phacoemulsification with intraocular lens implantation and received a topical with intracameral anesthetic. We attribute these successful outcomes to a standardized clinical workflow consisting of careful preoperative screening, communication and presence of VAD coordinator, continuation of antithrombotics, monitoring based on presence of pulsatile flow, and a plan for rapid transfer if needed.

## 1. Introduction

Left ventricular assist devices (LVADs) are being encountered more frequently in patients undergoing ophthalmic procedures but there is little information or experience with this patient population in the outpatient setting. LVADs provide mechanical circulatory support for patients with advanced heart failure. In the 1990s, LVADs were initially developed as a bridge to cardiac transplantation in patients with advanced cardiac failure [1]. As these devices have evolved over time, LVADs have been increasingly used as destination therapy [2]. Newer second generation devices, including the Heartmate II, use axial continuous flow for circulatory support [1]. Third generation devices, including HeartWare and DuraHeart, use centrifugal continuous flow [1]. Outcomes have been improved with the newer continuous flow LVADs [1]. The number of patients using these devices has increased accordingly and lifespans of these patients have also been increased [3]. Often, patients survive several years on these devices and develop noncardiac chronic diseases [4]. As overall quality of life improves for these patients, vision related quality of life should be addressed as well. Since quality of life is enhanced with improved vision, we present a case series of ophthalmic procedures performed on this challenging patient population.

## 2. Case Presentation

The University of Alabama's Institutional Review Board approved conduct of this study. Findings from the medical records of seven patients with LVADs undergoing 10 ophthalmic surgery procedures are summarized in Table 1. The median patient age was 72 years (range, 40-78). LVAD type was the Heartmate II (Thoratec, Pleasanton, CA). All patients were continued on their antithrombotic medications perioperatively. Cataract surgery performed under topical with intracameral anesthesia, without any sedation, was the most common scenario (8 cases). The longest case (59 min), phaco/IOL/vitrectomy/ERM peel, was performed with retrobulbar block on a patient taking uninterrupted Coumadin 2 mg qod and 3 mg qod with INR 1.8. One corneal case involving EDTA chelation, keratectomy, and amniotic membrane placement was performed under sub-Tenon's block on a patient taking uninterrupted Coumadin 3 mg qd, aspirin 325 mg qd, and plavix 75 mg qd with INR 1.5. Median surgical and operating room times were 12.5 mins (range, 6-59) and 35.5 mins (range, 29-82), respectively. Supplemental oxygen was administered to all patients. Noninvasive blood pressure (NIBP) monitoring was utilized without Doppler assistance in all patients. Pulse oximetry was utilized in all cases with a monitored waveform and oxygen saturation recorded. No cases of respiratory or hemodynamic compromise were noted and blood loss was negligible in all cases. No complications were noted during the perioperative time through same day discharge. All patients had an on-time discharge with no 30-day recidivism.

## 3. Clinical Workflow

The surgical procedures were coordinated through the University of Alabama's Heart Failure Program (HFP). A ventricular assist device (VAD) coordinator from the HFP familiar with the patient and their LVAD accompanied the patient to the ambulatory surgery unit and remained with the patient through discharge. The coordinator monitored flow, rpm, pulsatility index, and mean pressure on the flow console which they brought with them. The Heartmate II's have long lasting batteries and are smaller than previous generation LVADs which enhanced mobility. Our anticoagulation protocol for patients that received blocks (retrobulbar and sub-Tenon's) was to continue these medications as prescribed by cardiology throughout the perioperative period. We routinely block anticoagulated patients in our facility provided their therapies are not supratherapeutic. While the ambulatory facility did not house their own intensive care unit, X-ray capability, blood bank, or extensive lab capabilities, there was a process for rapid direct transfer to the University's Heart Failure Unit if needed. The patients were uniformly pleased to have their ophthalmic procedures performed and reported minimal discomfort.

Our series included seven LVAD patients who underwent 10 ophthalmic surgeries. Other reports in the literature cite the management of up to three LVAD patients having four cataract surgeries [5, 6]. In our experience, these procedures can be safely performed in a resource limited ambulatory facility, such as an ambulatory surgery center, provided a close working relationship with the VAD center is in place. The advantages of working with the VAD center include in-depth knowledge of the patients and their devices as well as a plan in place to facilitate rapid transfer to a higher acuity setting if needed. An advantage of utilizing an ambulatory center, provided this is where the clinician performs the vast majority of their cases, includes familiarity with staff, equipment, and supplies. The patients in this series all had enough pulsatile flow to allow use of NIBP monitors and pulse oximetry. In patients without pulsatile flow, other monitoring options exist. Options for blood pressure monitoring in patients without pulsatile flow include use of Doppler ultrasound while slowly deflating the blood pressure cuff, placement of an arterial catheter under ultrasound guidance, and intermittently turning down the flow on continuous flow devices to allow pressure measurement via oscillometry. For periodic oxygenation monitoring in patients without pulsatile flow, options include intermittently turning down the flow on continuous flow devices for pulse oximetry and arterial blood gas sampling from an arterial line. For continuous oxygenation monitoring in patients without pulsatile flow, options include use of cerebral oximetry, which does not require pulsatile flow, and continuous central venous saturation catheters. A monitoring plan should be in place for patients without pulsatile flow, particularly if surgery will take place at an ambulatory surgery center. While sedation may be helpful in some cases, it was not necessary in these highly motivated patients.

In summary, during the preoperative period, it is necessary to know the type of LVAD, to continue antithrombotics, and have the VAD coordinator on site. Intraoperatively, usual monitors may be used with pulsatile flow but Doppler ultrasound, arterial catheterization, cerebral oximetry, and intermittently decreasing device flow may be required in patients without pulsatile flow. Sedation is not always required. Postoperatively, patients are expected to go home, but a transfer plan to a higher acuity facility should be in place.

## Figures and Tables

**Table 1:** Patient characteristics and intraoperative data of patients with Left Ventricular Assist Device (LVAD) that underwent ophthalmic surgery.

Case	Age/gender	LVAD type	Procedure	Anesthesia	Sedation	Surgical time (mins)	OR time (mins)
1	72 /M	Heartmate II	Phaco/Iol	Topical+ intracameral	none	10	57
2	72 /M	Heartmate II	Phaco/Iol	Topical+ intracameral	none	16	53
3	73 /M	Heartmate II	Phaco/Iol	Topical+ intracameral	none	6	32
4	72 /M	Heartmate II	Phaco/Iol	Topical+ intracameral	none	17	32
5	59 /F	Heartmate II	Phaco/Iol	Topical+ intracameral	none	10	34
6	59 /F	Heartmate II	Phaco/Iol	Topical+ intracameral	none	6	30
7	40 /F	Heartmate II	EDTA scrub/keratectomy/AMT	Sub-Tenon's block	none	22	48
8	78 /M	Heartmate II	Phaco/Iol/vitrectomy/IVA/ERM peel	Retrobulbar block	none	59	82
9	78 /M	Heartmate II	Phaco/Iol	Topical+ intracameral	none	8	29
10	52M	Heartmate II	Phaco/Iol	Topical+ intracameral	none	15	37

LVAD: left ventricular assist device, M: male, F: female, Phaco/lol: phacoemulsification with intraocular lens implantation, EDTA: ethylenediamine-tetraacetic acid, AMT: amniotic membrane transplantation, IVA: intravitreal avastin, and ERM: epiretinal membrane.
